# The role of navigation bronchoscopy in the workup of lung lesions in lung transplant-eligible patients: A single-center case series

**DOI:** 10.1016/j.jhlto.2026.100614

**Published:** 2026-06-18

**Authors:** R. van Pel, R. Cornelissen, A. Duininsveld, B.J. Mathot, L. Seghers, K.I.M. Looman, J.G.J.V. Aerts, M.E. Hellemons

**Affiliations:** a Department of Respiratory Medicine, Erasmus University Medical Center, Rotterdam, the Netherlands; b Erasmus MC Transplant Institute, Erasmus University Medical Center, Rotterdam, the Netherlands

**Keywords:** Lung transplant, Nodule, Bronchoscopy, Navigation bronchoscopy, Intervention bronchoscopy

## Abstract

**Introduction:**

Lung transplantation (LTx) eligibility assessment may reveal lung lesions suspicious for malignancy. Histological evaluation of these lesions may provide clarity regarding their origin and prevent delays in transplant eligibility decisions. This single-center case series presents the outcomes of navigational bronchoscopy in patients undergoing evaluation for LTx.

**Methods:**

All patients evaluated for LTx between September 2023 and December 2025 with suspicious lung lesions amenable to navigational bronchoscopy with cone-beam CT and navigation software were included in this retrospective single-center study.

**Results:**

Out of 173 patients evaluated for lung transplantation, twelve (7%) had suspicious lung lesions. Eight patients were considered eligible for navigational bronchoscopy. The median nodule size was 12 mm (range 6–24 mm), with Herder risk scores ranging from 13% to 84% probability of malignancy. Seven patients had COPD as the underlying disease, with a median FEV₁ of 23% and DLCO of 35%. One patient had chronic lung allograft dysfunction. In all cases, the nodules were successfully reached. No complications occurred related to bronchoscopy or general anesthesia. The median procedure time was 60 min, and the median hospital stay was 7 h. In 88% of cases, the pathological results had direct clinical consequences for transplant eligibility and subsequent management.

**Conclusion:**

This series demonstrates that navigational bronchoscopy is a safe and effective diagnostic tool for evaluating suspicious lung lesions in patients undergoing assessment for lung transplantation. It can safely accelerate transplant eligibility decision-making and reduce diagnostic uncertainty.

## Introduction

Lung transplant (LTx) can be a lifesaving treatment for end-stage lung disease.^1^ To assess feasibility, and due to the shortage of donor lungs, a strict pre-transplant evaluation process is performed.^2^

During this evaluation, pulmonary nodules of uncertain etiology are commonly detected on chest computed tomography (CT). Reported prevalences exceed 7% for suspicious lung lesions on pre-transplant chest CT, most commonly in former smokers with chronic obstructive pulmonary disease (COPD).^3^

Based on medical history, radiological nodule characteristics, positron emission tomography (PET)-CT findings, and interval progression, an assessment of malignancy risk is made, incorporating risk-stratification tools such as the Herder score.^4^ Active (lung) malignancy is an absolute contraindication for lung transplantation,^2^ and depending on the tumor type, a significant disease-free interval is generally required before transplantation can be reconsidered.^5^ Consequently, when incidental suspicious nodules of unknown significance are detected, patients may be rejected, (temporarily) suspended, or delisted from the transplant waiting list.

Notably, a recent study in a high-volume lung transplant center demonstrated that 7.4% of the patients were listed with a pulmonary nodule without knowledge of its etiology. A small proportion of these cases (15%) were ultimately found to be malignant. The majority of these patients had COPD (83.5%).^3^

Obtaining histologic diagnosis is often considered unattainable in this high-risk population with end-stage lung disease. Transthoracic needle biopsy, the most commonly used diagnostic procedure, carries significant risks, with reported pneumothorax and hemorrhage rates of up to 30% in this population.^6^ As such, the ultimate decision to reject or accept a candidate for LTx is often made based on incomplete information. A reliable and safe diagnostic tool for lesion characterization in high-risk patients is urgently needed to avoid unjustified exclusion from LTx and accelerate transplant eligibility decision.

With the advent of new techniques, such as cone beam computed tomography (CBCT),^7^ navigational bronchoscopy has become considerably more reliable and precise. Diagnostic yield is currently comparable to transthoracic biopsy, with substantially lower risks.^8^, ^9^ Moreover, it has already been shown that electromagnetic navigational bronchoscopy is safe even in patients with moderate to severe COPD.^10^

The diagnostic yield of navigational bronchoscopy can be classified according to Vachani et al.^11^ into three categories: strict (only immediate malignant or specific benign diagnoses), intermediate (including non-specific benign findings confirmed on follow-up), and liberal (additionally including initially non-diagnostic samples confirmed as benign on follow-up).

With this case series, we aim to highlight the potential clinical value of navigational bronchoscopy in reducing diagnostic uncertainty and accelerating decision-making in lung transplant candidates, a setting in which data remain scarce and awareness is limited. We present the first real-world series on the use of CBCT-guided navigational bronchoscopy in LTx candidates with suspicious lung lesions.

## Methods

This study was a single-center retrospective case series. Navigational bronchoscopy with cone-beam CT was introduced at our institution in September 2023. From that time until December 2025, all patients undergoing evaluation for lung transplantation with a suspected pulmonary nodule were systematically assessed for the feasibility of obtaining histological confirmation via bronchoscopy.

Each patient underwent PET-CT and malignancy risk stratification using the Herder risk model. In addition, to assess disease severity and general anesthesia risk cardiac status was evaluated, including right heart catheterization to determine the presence of pulmonary hypertension. For each case, a multidisciplinary assessment was conducted with the lung transplant consultant, interventional pulmonologist, and anesthesiologist, with particular attention paid to the probability of success and the risk for complications*.* Procedures were performed on an outpatient basis unless admission was deemed necessary for observation. Each patient was routinely administered a course of prednisolone to avoid post-procedural exacerbation in line with national guideline.

All patients had provided written informed consent for transplant-related research, and the study was approved by the local ethics committee (METc CMO 2019–5148).

Navigational bronchoscopy was performed under general anesthesia by a dedicated team of three interventional pulmonologists. Navigation software (Synapse 3D, Fujifilm, Japan) was used for guidance to the target lesion, and radial endobronchial ultrasound (rEBUS, Fujifilm, Japan) was used as tool to confirm the correct position of the sheath in the lesion. Cone-Beam CT was performed to generate 3D representations of the lesion and the navigation pathway, confirming correct position. Augmented fluoroscopy was subsequently used to overlay these images with real-time two-dimensional fluoroscopy. Routinely, initial cytology was obtained using needle aspiration, followed by histological sampling by a forceps biopsy. Rapid on-site evaluation (ROSE) was routinely performed by a cytologist for biopsy assessment. If ROSE was negative, cryobiopsy was considered using a 1.1 mm cryoprobe (Erbe, Elektromedizin GmbH, Germany) with a freezing time of 4–8 s. Clinical impact was defined as a bronchoscopic finding that directly influenced or accelerated transplant decision-making by reducing multidisciplinary team (MDT)-assessed residual malignancy risk, obviating the need for additional diagnostic procedures and delay waiting list by follow up and thus directly determining transplant eligibility (listing, transplantation, or exclusion). All analyses were conducted with IBM SPSS Statistics version 24 (IBM, NY, USA). Continuous data are expressed as median and range. Variables are expressed as percentages.

## Results

**Figure 1:**
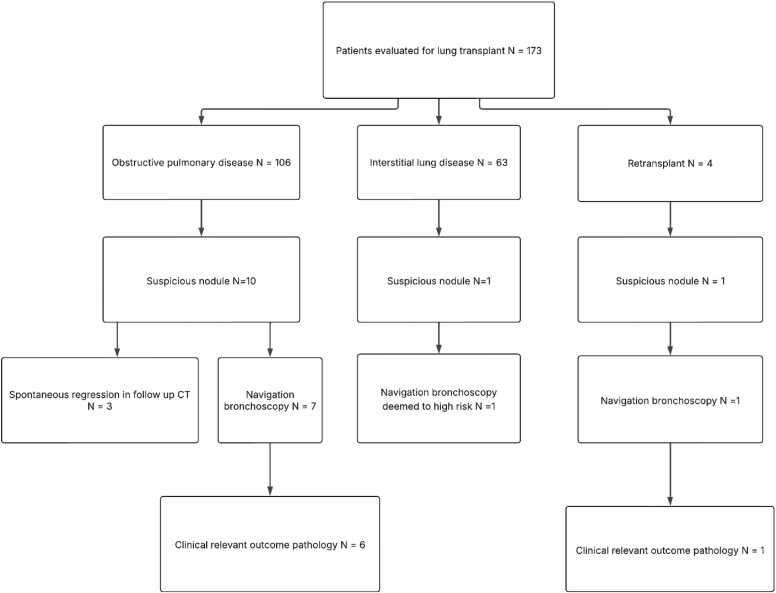
Patient selection.

Within the study time frame, 173 patients were evaluated for LTx. Of these, 106 (61%) had obstructive pulmonary disease, 63 (36%) had interstitial lung disease (ILD), and 4 underwent re-transplantation for chronic lung allograft dysfunction (CLAD). Figure one highlights the patient selection. Suspicious pulmonary nodules were detected in 12 patients (7%). In all patients, these nodules were new compared with the most recent CT scan performed at the referring center. In three cases, spontaneous regression of the nodule was detected at first follow-up, and no additional diagnostic tests were performed. Nine out of 173 evaluated patients (5.2%) were considered for navigation bronchoscopy (Figure 1). In one navigational bronchoscopy was considered too high risk, primarily due to risks associated with general anesthesia. This patient had severe pulmonary hypertension (mPAP 48 mmHg) and very severely reduced diffusing capacity of the lung for carbon monoxide corrected for hemoglobin (DLCOc 11*%).* Eight patients (4.6%) had a single suspicious pulmonary nodule accessible for navigational bronchoscopy. The median time from referral to bronchoscopy was 17 days (range 12–37 days). Patient characteristics of these patients are shown in Table 1. Median age was 61 years (range 50–65 years); four patients (50%) were female. All but one patient had COPD as underlying disease; one patient was evaluated for CLAD. For the patients with COPD, the median forced expiratory volume in one second (FEV1) was 23% (range 19–25%), the median forced vital capacity (FVC) was 61% (range 51–99%), and the median DLCOc was 35% (range 26–54%). Of the eight patients assessed, three required supplemental oxygen at rest (ranging from 1 to 4 L/min). All patients required supplemental oxygen during exertion, with flow rates ranging from 1 to 5 L/min. All patients received a five-day course of prednisolone 30 mg prior to bronchoscopy. Herder scores ranged from 13% to 84% for risk of malignancy. Lesions were located in the right upper lobe (N=3), left upper lobe (N=3), right lower lobe (N=1), and left lower lobe (N=1). Median nodule size was 12 mm (12 mm (range 6–24 mm)). In all cases, a bronchus sign was visible on the CT scan. In 7 patients (88%), the nodule was located in the outer third of the thorax. The median mean pulmonary artery pressure (mPAP) was 18 mmHg (range 10–29 mmHg).

**Table 1:** Characteristics of the Study Population

Patient no	1	2	3	4	5	6	7	8
Sex	F	M	M	F	F	F	F	M
Age (years)	61	55	60	57	65	63	62	50
Location nodule	RUL	RUL	LUL	RLL	LLL	RUL	LUL	RUL
Periphery of Middle 1/3 thorax	Middle	Peripheral	Peripheral	Peripheral	Peripheral	Peripheral	Peripheral	Peripheral
PET-CT FDG uptake	Moderate	High	Moderate	Faint	Moderate	Intens	Moderate	Moderate
Herder prediction model score (%)	57	70	68	13	56	84	57	60
Size (mm)	6	16	12	14	10	12	6	24
Nodule aspect on CT thorax	Solid	Solid	Solid	Solid	Solid	Solid	Solid	Solid
Bronchus sign	Yes	Yes	Yes	Yes	Yes	Yes	Yes	Yes
Duration of bronchoscopy (minutes)	35	67	54	54	60	72	60	60
Duration admittance (hours)	5	15	6	7	28	8	7	7
Radiation dose (Dose area product in mGy·m²)	0	1613	1557	883	2362	1186	2187	4995
Cone beam CT spins	0	2	1	1	2	2	1	2
Indication diagnosis LTx	COPD	COPD	COPD	COPD	COPD	COPD	COPD	CLAD
FEV1 in liters (% pred)	0.62 L (23%)	0.86 L (24%)	0.88 L (25%)	0.42 L (19%)	0.54 L (23%	0.53 L (22%)	0.44 L (21%)	1.17 L (29%)
FVC in liters (% pred)	1.89 L (55%)	4.55 L (99%)	4.62 L (99%)	1.68 L (61%)	1.93 L (66%)	1.81 L (56%)	1.32 L (51%)	1.770 L (35%)
DLCO (% pred)	35%	26%	34%	33%	54%	47%	46%	52%
mPAP (mmHg)	26	29	17	26	19	17	16	24

Abbreviations: F, female; M, male; RUL, right upper lobe; LUL, left upper lobe; LLL, left lower lobe; PET, positron emission tomography, CT, Computed tomography; COPD, Chronic obstructive pulmonary disease; CLAD, Chronic lung allograft dysfunction; LTx, lung transplant; FEV1, forced expiratory volume 1 s; FVC, forced vital capacity; DLCO,diffusion capacity of the lung for carbon monoxide; mPAP (mean pulmonary arterial pressure measured by right heart catheterization

Median procedure time was 60 min (range 35–72 min). No complications occurred during or after bronchoscopy. There were no cases of pneumothorax, significant bleeding, or anesthesia-related issues. Median hospital stay was 7 h (range 5–28 h). Cytology and histology samples were obtained in all procedures. Cryobiopsy was used in two cases.

Using strict diagnostic criteria, a definitive diagnosis was established in 3 of 8 (38%) cases at the time of bronchoscopy. When 6 months of clinical follow-up were incorporated, the intermediate diagnostic yield increased to 7 of 8 (88%) without evidence of subsequent diagnostic revision or need to alter management.

**Figure 2:**
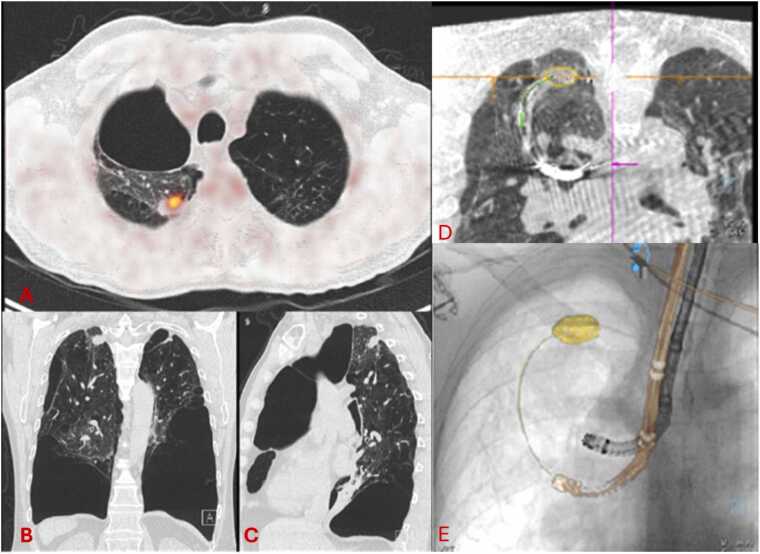
A; PET-CT scan of patient 2 in axial plane with FDG-avid hotspot in right upper lobe. B and C; CT scan of patient 2 in coronal and sagittal plane with nodule in right upper lobe with pronounced bullous emphysema. D: Cone beam CT image with colored lesion in right upper lobe (Yellow) and endobronchial route (Green) E: overlay augmented fluoroscopy during bronchoscopy to reach nodule in right upper lobe.

The relationship between bronchoscopic findings, MDT-assessed malignancy risk, and transplant decision-making at the individual patient level is shown in Table 2. Navigational bronchoscopy influenced or accelerated transplant decision-making in 7/8 patients (88%). In patients with specific pathological diagnoses, such as adenocarcinoma (Patient 6), plant material with Actinomyces and fungal elements interpreted as aspiration (Patient 1), or chronic rejection (Patient 8), bronchoscopic findings directly established a definitive diagnosis. In patients with non-specific inflammatory or reactive findings (Patients 2, 3, 4, and 7) (Figure 2), bronchoscopy contributed to MDT-based risk stratification by lowering or contextualizing malignancy probability. Although these findings did not definitively exclude malignancy, they reduced diagnostic uncertainty sufficiently to avoid additional invasive testing or prolonged surveillance. In one patient with atypical cells (Patient 5), malignancy could not be excluded, and additional VATS wegde resection definitively excluded malignancy. This patient is currently excluded from transplant for other medical reasons unrelated to procedures.

**Table 2:** Patient-level Relationship Between Bronchoscopic Findings, Multidisciplinary Team (MDT) Interpretation of Residual Malignancy Risk, and Subsequent Transplant Decision-making

Patient no	1	2	3	4	5	6	7	8
Pathology and microbiology	Plant residue and fungus Presence of *Actinomyces*, plant material, and fungal hyphae.	Active inflammation and eosinophilia	Lung parenchyma and bronchial tissue with reactive changes, alveolar macrophage and anthracosis.	Chronic inflammation with mild fibrosis and focal eosinophilia + gram positive culture.	Atypical cells	Adenocarcinoma	Chronic inflammation, anthracosis and fibrosis.	Chronic rejection
MDT interpretation of residual malignancy risk	Excluded	Low	Low	Excluded	High	Definite malignancy	Low	Excluded
Expected management without bronchoscopy	Rejection, TTNA not possible	6 months delay waiting list, repeat imaging and possible TTNA	6 months delay waiting list, repeat imaging and possible TTNA	6 months delay waiting list, repeat imaging and possible TTNA	TTNA followed by VATS wedge resection	6 months Follow-up imaging or TTNA followed by rejection	6 months delay waiting list, repeat imaging and possible TTNA	Rejection, TTNA not possible
Additional diagnostics avoided	Yes	Yes	Yes	Yes	No	Yes	Yes	Yes

Abbreviations: MDT, multidisciplinary team; TTNA, transthoracic needle aspiration

## Discussion

This retrospective case series describes eight patients with end-stage lung disease who underwent navigational bronchoscopy after detection of a suspicious incidental pulmonary nodule on CT and PET imaging during LTx eligibility assessment. Such incidental pulmonary nodules frequently present a significant clinical dilemma in candidates evaluated for LTx. This case series provides the first real-world evidence that navigational bronchoscopy has a role specifically in this population. We demonstrated that navigational bronchoscopy can be performed safely in LTx candidates and that this approach has clear and direct clinical implications, including earlier establishment of a pathological diagnosis, reduction of diagnostic uncertainty, and avoidance of or unwarranted exclusion from the transplant waiting list. Despite 88% of the nodules located in the outer one-third of the thorax, no complications occurred such as a pneumothorax. This is in line with recent studies covering navigation bronchoscopies which show very low complication rates as well.^8^

In all cases described, the detected lesions represented newly identified abnormalities for which no prior longitudinal imaging was available. Although the strict definition of diagnostic yield is generally preferred, its application in this population may be limited. In patients with COPD, non-specific inflammatory changes are frequently encountered, complicating the interpretation of histopathological findings.^12^ Therefore, clinical decision-making should integrate all available data, including imaging characteristics, procedural findings, and clinical context. Notably, in this case series, no interval changes on follow-up CT imaging led to revision or reversal of the initial clinical management decisions. According to the intermediate classification, 7 of 8 (88%) patients had findings that were ultimately confirmed as benign after six months of follow-up.

In the context of lung transplantation, the primary clinical objective extends beyond establishing a definitive diagnosis of malignancy; rather, it centers on accurate risk stratification to enable timely and safe listing decisions. A high pre-test probability of malignancy may delay listing and necessitate a period of surveillance. This delay is clinically significant, given the considerable risk of disease progression or waitlist mortality in this vulnerable population.

Patients were carefully selected, all presenting with severe lung disease but otherwise stable cardiac function without significant pulmonary hypertension (mPAP <30 mmHg). No complications occurred in any case, either related to the bronchoscopy or anesthesia, and with a short admission time, most patients could be treated in an outpatient setting. A single patient was admitted due to practical logistical reasons.

Inclusion of patients was based on (1) the clinical indication for histological diagnosis in the context of lung transplant evaluation, (2) radiological characteristics suggesting malignancy, and (3) procedural feasibility and safety. Exclusion criteria include lesions with very low pre-test probability of malignancy, lesions unlikely to be reachable through bronchoscopy, or cases in which the procedural risk (e.g., due to comorbidities or anesthesia risk) outweighs the expected clinical benefit. Following screening and while on the waiting list, all patients undergo routine annual CT scanning for the early detection of pulmonary nodules. In this specific patient subgroup, the imaging interval is shortened to 3–6 months, depending on the individual case. In the current case series, all included lesions were solid nodules with 7/8 (88%) of patients with underlying COPD, reflecting the cases encountered during the study period rather than a predefined restriction of the technique.

In our practice, lesion selection for navigational bronchoscopy with cone-beam CT is not limited to solid nodules. Ground-glass nodules or FDG-PET negative lesions may also be considered, provided there is sufficient suspicion of malignancy and a reasonable likelihood that tissue diagnosis will impact clinical decision-making.

Despite 36% of the screened patients having underlying interstitial lung disease (ILD), no patients in this cohort with ILD were identified with a pulmonary nodule in this cohort. Nevertheless, the procedure may still be applicable in patients with ILD.

However, attention should be given to potential severe pulmonary hypertension and the risk of lesion disappearance on CBCT due to atelectasis associated with positive-pressure ventilation. There is limited evidence on ILD exacerbations following navigational bronchoscopy. However, data from transbronchial cryobiopsy—using a similar approach—suggest a negligible risk of post-procedural exacerbations.^13^

While navigational bronchoscopy may not be suitable for all patients, our series demonstrates that this approach is feasible and can aid decision-making in a substantial proportion of patients.

This series provides valuable insights for transplant professionals by raising awareness of the possibilities and potential benefits of this technique in a highly complex patient population and setting. Larger studies with longer follow-up and standardized endpoints are needed to further validate these observations.

## Declaration of Competing Interest

The authors declare that they have no known competing financial interests or personal relationships that could have appeared to influence the work reported in this paper.
